# Sound Localization Bias and Error in Bimodal Listeners Improve Instantaneously When the Device Delay Mismatch Is Reduced

**DOI:** 10.1177/23312165211016165

**Published:** 2021-05-31

**Authors:** Julian Angermeier, Werner Hemmert, Stefan Zirn

**Affiliations:** 1 Peter Osypka Institute of Medical Engineering, Faculty of Electrical Engineering, Medical Engineering and Computer Sciences, University of Applied Sciences Offenburg, Germany; 2 Bio-Inspired Information Processing, Munich School of Bioengineering, Technical of University Munich, Germany

**Keywords:** bimodal hearing, sound localization, interaural stimulation timing, cochlear implant, hearing aid

## Abstract

Users of a cochlear implant (CI) in one ear, who are provided with a hearing aid (HA) in the contralateral ear, so-called bimodal listeners, are typically affected by a constant and relatively large interaural time delay offset due to differences in signal processing and differences in stimulation. For HA stimulation, the cochlear travelling wave delay is added to the processing delay, while for CI stimulation, the auditory nerve fibers are stimulated directly. In case of MED-EL CI systems in combination with different HA types, the CI stimulation precedes the acoustic HA stimulation by 3 to 10 ms. A self-designed, battery-powered, portable, and programmable delay line was applied to the CI to reduce the device delay mismatch in nine bimodal listeners. We used an A-B-B-A test design and determined if sound source localization improves when the device delay mismatch is reduced by delaying the CI stimulation by the HA processing delay (τ_HA_). Results revealed that every subject in our group of nine bimodal listeners benefited from the approach. The root-mean-square error of sound localization improved significantly from 52.6° to 37.9°. The signed bias also improved significantly from 25.2° to 10.5°, with positive values indicating a bias toward the CI. Furthermore, two other delay values (τ_HA_ –1 ms and τ_HA_ +1 ms) were applied, and with the latter value, the signed bias was further reduced in some test subjects. We conclude that sound source localization accuracy in bimodal listeners improves instantaneously and sustainably when the device delay mismatch is reduced.

Bimodal stimulation for cochlear implant (CI) users has become a common approach. In such cases, one ear is provided with a CI, and the contralateral ear receives a conventional digital hearing aid (HA). Many studies have been published showing a benefit for most bimodal listeners in binaural performance, when both devices were used instead of just one (Ching et al., 2004, 2006; Hoppe et al., 2018; Sheffield et al., 2017), along with an improvement in quality of life (Farinetti et al., 2015). Despite the reported benefits, Dorman et al. (2016) showed that in terms of sound source localization, bimodal CI/HA users performed more poorly than bilateral CI users and bilateral HA users.

The binaural cues for sound localization in the horizontal plane are interaural level differences (ILDs) and interaural time differences (ITDs) in the temporal envelope and fine structure. ILDs are most prominent at frequencies greater than 1500 Hz. Sound information at such high frequencies is well transmitted with the CI but is often barely audible with the HA because of limited residual hearing (Hoppe et al., 2018). Thus, due to little spectral overlap in both ears, ILD perception may be hampered in many bimodal listeners (Dorman et al., 2015; Seeber et al., 2004). But even with limited high-frequency residual hearing at the HA side, the perception of envelope ITDs may still be possible. Dirks et al. (2020) showed in single-sided deaf (SSD) subjects that binaural beats are perceivable with electric/acoustic stimulation over a wide frequency range. Further Francart et al. (2009) could measure ITD just-noticeable differences (JNDs) for bimodal subjects in direct stimulation experiments with adjusted interaural stimulation timings at acoustic frequencies above 1 kHz, suggesting sensitivity to envelope ITDs. On the other hand, ITDs conveyed in the temporal fine structure of the ear signals are likely to be not perceivable by bimodal listeners because interaural phase information is typically not provided by current CI coding strategies (Zirn et al., 2016).

Another, yet rarely discussed, problem faced by bimodal listeners is that the two different hearing devices may have very different processing delays. Zirn et al. (2015) showed that there can be a temporal delay in the range of 3 to 10 ms between an ear provided with a MED-EL Maestro CI system and the contralateral ear provided with a HA. In the ear provided with the CI, the frequency-dependent latencies arise from the signal processing by the speech processor and have been shown to be relatively close to latencies occurring in the normal-hearing ear in MED-EL Maestro CI systems. In an ear provided with an HA, the absolute latency is a combination of the (mostly) frequency-independent HA processing latency and the physiologically occurring latencies arising from the transmission of sound through outer, middle, and inner ear, where a frequency-dependent latency component is added due to the basilar-membrane travelling wave delay. This temporal asymmetry between the modalities is further referred to as device delay mismatch and may, if present, hamper the perception of envelope ITD and ILD. In the latest study by Zirn et al. (2019), a significant improvement in sound source localization ability was reported for bimodal CI/HA users, when the device delay mismatch was minimized by delaying the CI stimulation. An unresolved question so far is whether the measured HA delay ( $t_{\mathrm{Delay}}$ = *τ_HA_*) is the optimal value to compensate the device delay mismatch. Figure 1 shows three different delay values for the CI stimulation (*τ_HA_*–1 ms, *τ_HA_* and *τ_HA_*+1 ms), which were applied in the current study. The latency curves reveal the resulting temporal overlap. The motivation to use these three values for $t_{\mathrm{Delay}}$ was based on the findings of Zirn et al. (2015). According to this previous work, *τ_HA_*–1 ms leads to a good temporal adjustment of the modalities at lower frequencies (0.5–1 kHz), *τ_HA_* in the middle frequency range (1–2 kHz), and *τ_HA_*+1 ms at higher frequencies (2–4 kHz). The resulting frequency-dependent latencies are relatively close but do not perfectly match the latencies of an ear provided with a HA.

**Figure 1.**
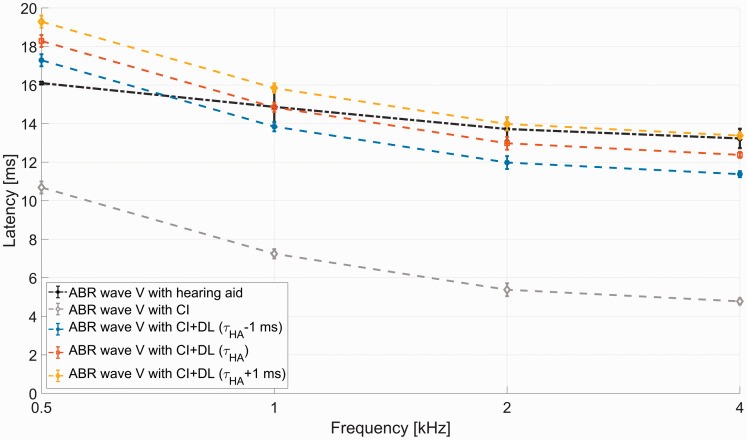
Effects of the Three Applied Delays for Device Delay Mismatch Reduction on ABR Wave V Latency With a CI Compared to ABR Wave V Latency With a Phonak Una M HA (*τ_HA_* = 7 ms) Showing the Temporal Adjustment in the Different Frequency Ranges for the Three Applied Values for *τ_HA_*. Adapted from Zirn et al. (2015). ABR = auditory brainstem response; CI = cochlear implant; DL = delay line.

**Figure 2.**
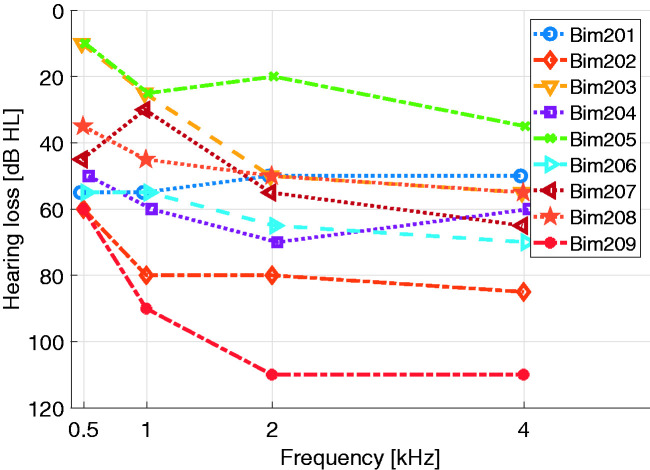
Residual Hearing of all Subjects Included in this Study on the Ear Provided with HA.

Evidence that the 1-ms stepsize around *τ_HA_* makes a difference comes from Seebacher et al. (2019), who could show an improvement in sound localization if the CI stimulation was delayed by 1 ms in SSD CI users leading to a lower device delay mismatch in the corresponding high-frequency band (2–4 kHz).

Compared with Zirn et al. (2019), the A-B study design was extended to an A-B-B-A design in the present work. In the former A-B study design, localization tests were conducted without minimizing the device delay mismatch (Condition A) and after a familiarization period of 1 hr with the minimized device delay mismatch (Condition B). In the current study design, localization tests were conducted without minimizing the device delay mismatch (first test in Condition A), acutely after minimizing the device delay mismatch (first test in Condition B), after 1 hr of familiarization to the changed delays (second test in Condition B), and acutely after resetting the device delay mismatch to its initial value (second test in Condition A). This allows us to investigate the effects of the familiarization as well as ruling out procedural training over the course of the study. Furthermore, not only the root-mean-square (rms) error is analyzed but also the signed bias, which delivers information about the direction of the localization error. Further additional details improved the study design, and all changes are described in the methods section.

## Material and Methods

### Test Subjects

Nine adult bimodal listeners (age: 61.1 ± 6.9 years, min. 47 years, max. 71 years; 3 females, 6 males) participated in the study. Details are listed in Tables 1 and 2. None of the subjects had participated in earlier studies.

**Table 1.** Data of Bimodal Subjects.

Subject	Age (years)	Aetiology	CI type (processor/implant)	Implanted side	CI and bimodal experience (years)	HA experience (years)	CI coding strategy
Bim201	56	Progressive	RONDO2/CONCERTO Flex28	left	1	5	FS4
Bim202	71	Acute hearing loss	OPUS2/CONCERTO Flex 28	right	8.5	29	FS4
Bim203	61	Blast trauma	RONDO2/SYNCHRONY Flex28	right	2	6	FS4
Bim204	59	Sudden hearing loss	SONNET/SYNCHRONYFlex28	left	2	9	FS4-p
Bim205	64	Acute hearing loss	SONNET/SYNCHRONY Flex28	right	5.5	4	FS4
Bim206	66	Unknown	SONNET EAS/SYNCHRONY Flex28	right	2	3	FS4
Bim207	58	Progressive	RONDO/SYNCHRONY Flex28	left	6	11	FS4
Bim208	68	Meniére	SONNET/SYNCHRONY Flex28	right	2	7	FS4-p
Bim209	47	Unknown	SONNET/SYNCHRONY Flex28	left	5.5	18	FS4

*Note*. CI = cochlear implant; HA = hearing aid.

**Table 2.** Hearing Aids of the Bimodal Subjects and Average Processing Delays (*τ_HA_*) of These Devices.

Test subject	HA type	Averaged *τ_HA_* (ms)
Bim201	ReSound LiNX2 LS9	5.9 (min: 4.3; max: 8.3)
Bim202	Widex Daily 100 Fashion	3.9 (min: 2.6; max: 5.6)
Bim203	Bernafon IN1 N	5.8 (min: 5.5; max: 6)
Bim204	Widex Evoke 220 Fa P	2.8 (min: 1.7; max: 5.3)
Bim205	Widex Daily 50 D-FA	2.8 (min: 2.1; max: 3.0)
Bim206	Widex Beyond 330 B3-F2	2.8 (min: 1.4; max: 5.4)
Bim207	Oticon Selectic Napoli Pro	6 (min: 5.8; max: 7.1)
Bim208	Oticon NovaSense Geneve	5.2 (min: 4.9; max: 5.5)
Bim209	Phonak Naida Q90 UP	7.2 (min: 6.7; max: 7.7)

*Note*. HA = hearing aid.

On the ear provided with the HA, the subjects had mild to severe sensorineural, conductive, or mixed hearing losses (see Figure 1 for air conduction pure tone thresholds). Criteria for inclusion of bimodal subjects in the study were (a) everyday use of their HA and CI and (b) a percent correct score of more than 50% obtained in the Freiburg monosyllabic (word) test (Hahlbrock, 1953) at 65 dB sound pressure level (SPL) in a free field measurement (both on the CI and HA side monaural as well as binaural). The test subjects had no residual acoustic hearing in the ear provided with the CI at the frequencies 0.5, 1, 2, and 4 kHz. All subjects had complete insertions except for Bim206, where the 12th electrode was outside the cochlea and the 11th and 12th electrodes were deactivated due to high impedances. All testing was conducted in accordance with the Code of Ethics of the World Medical Association (Declaration of Helsinki) for experiments involving humans. Approval by the Technical University of Munich ethics committee was obtained (340/19). All subjects provided written informed consent.

### The Delay Line

As the subjects used their own HAs in this study, the HA delay (*τ_HA_*) was measured for every individual HA the test subjects came with, using either the HA analyzer unit ACAM 5 from Acousticon GmbH, Reinheim, Germany, as described in Zirn et al. (2015), or a self-constructed measuring setup, which delivers similar values. In this setup, a white noise burst (100 Hz–20 kHz) at a level of 65 dB (A) was presented to a reference microphone (Behringer ECM 8000) and the HA microphone. The sound tube of the HA was connected to a measurement microphone, identical to the reference microphone, via a 2 ccm coupler. To calculate frequency-dependent delays, a digital zero-phase bandpass filterbank with center frequencies of 500 Hz to 6 kHz and bandwidths of 500 Hz was implemented in MATLAB. After bandpass filtering, the frequency-specific delay was calculated for each band via cross correlation of the HA signal and the reference signal. In case of high-frequency hearing loss, only the delays at frequencies where the hearing loss was ≤ 80 dB HL were considered for averaging. The corresponding *τ_HA_* values can be found in Table 2.

To delay sound signals at the CI with sufficient temporal resolution in bimodal listeners, a programmable delay line (DL) based on the Arduino Due microcontroller (µC) board with a built-in Atmel SAM3X8E ARM Cortex-M3 CPU was used. The DL corresponds exactly to the one used and described in detail in Zirn et al. (2019). With a sampling frequency of 48 kHz, it provides the possibility to delay signals by integer multiples of 20.8  µs while ensuring a very low minimum delay of only 50 µs. The delay applied between analog-to-digital (AD) and digital-to-analog (DA) conversion is based on a ring buffer and is therefore frequency-independent. Both analog-to-digital converter (ADC) and digital-to-analog converter (DAC) have a 12-bit resolution.

The only difference to the DL used in the study from 2019 is the type of power supply. Whereas a 9 V rechargeable battery was used earlier, a 2,500 mAh lithium-ion battery combined with a step-up voltage regulator was used to provide power to the DL. With this type of rechargeable battery, weight of the DL could be reduced, and the runtime extended.

In the present study, the time by which the CI stimulation was delayed ( $t_{\mathrm{Delay}}$ ) was set to *τ_HA_* (see Table 2) which was considered as an estimate for the device delay mismatch (i.e., *τ_HA_* ≈ device delay mismatch). This was decided based on the results presented by Zirn et al. (2015) in which the latencies of the CI stimulation in MED-EL CIs (where the coding strategy uses a filter bank) were comparatively close to the physiological delays introduced mainly through the travelling wave delay on the basilar membrane. In a subset of the test subjects (Bim203, Bim204, Bim205, Bim206, Bim207, Bim208, Bim209), $t_{\mathrm{Delay}}$ was also set to *τ_HA_*–1 ms and *τ_HA_* + 1 ms to evaluate if *τ_HA_* is really an appropriate value by which to delay the CI stimulation.

The DL is inserted into the signal path of the CI as follows: An OPUS2 CI audio processor, worn behind the ear, was used to capture the acoustic signal. This unprocessed signal was fed into the DL where it was delayed. After the delay was applied, the signal was fed into another OPUS2 CI audio processor programmed with the same settings as the subjects’ everyday program. Further in this second OPUS2, the microphones were internally switched off. For further details, see Zirn et al. (2019), Figure 3.

**Figure 3.**
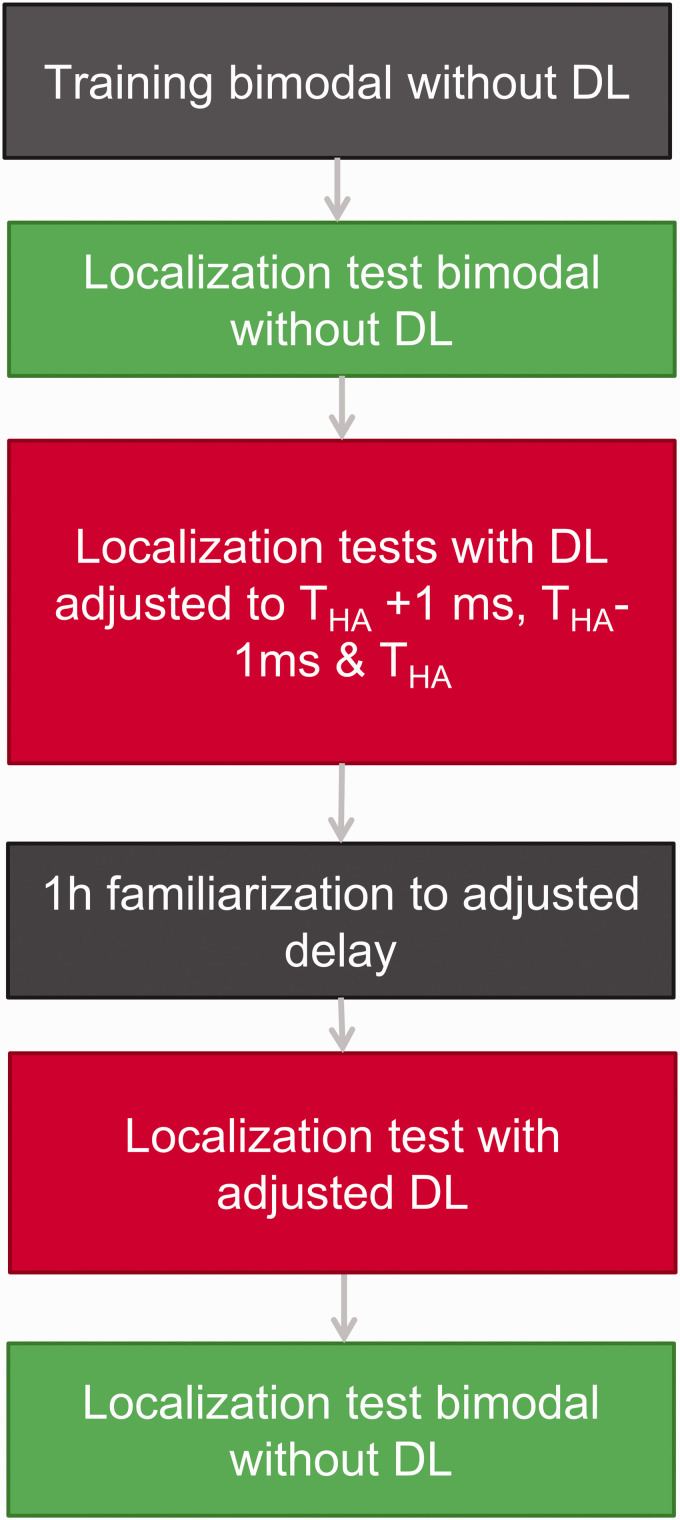
Schematic of the A-B-B-A Test Design (A = Green, B = Red). DL = delay line.

### Test Environment

All tests were conducted in the same audiometric booth as for the earlier study published (Zirn et al., 2019). Seven loudspeakers (type Genelec 8030 C) were located at an angular spacing of 30° between –90° (loudspeaker #1) to 90° (loudspeaker #7) in a semicircle in the frontal horizontal plane at the subject’s head level with 1 m between the subject’s head and each loudspeaker. The loudspeakers carried number plates from 1 to 7. The study participants used an app running on a tablet computer depicting the loudspeaker arc with the numbers of the speakers to type in their responses. These responses were sent to a personal computer outside the audiometric booth via Bluetooth where the data were then processed in MATLAB.

### Stimuli

In this study, we presented multiple noise bursts as stimuli in the sound source localization experiments. Stimuli were generated in MATLAB (The Mathworks Inc., Natick, MA, USA) and consisted of five Gaussian white noise bursts (125 Hz to 20 kHz). Bursts had a duration of 70 ms with 3 ms Gaussian-shaped slopes and were separated by 30-ms pauses. A similar type of stimulus was used by Seeber et al. (2004) in a study involving bimodal listeners. To avoid the use of monaural cues in the localization task, spectral roving and level roving was applied to the stimuli for each trial. Level roving was achieved by randomly presenting stimuli at 60, 65, or 70 dB (A). For spectral roving, the stimulus was filtered either by the ipsilateral or contralateral HRTF taken from an open HRTF database (Kayser et al., 2009) for a stimulus azimuth of 90° according to Van de Heyning et al. (2016). This led to a total of 42 (7 loudspeakers × 3 levels × 2 spectra) different combinations.

### Experimental Procedure

The complete measurement procedure is illustrated in Figure 3.

Prior to the localization tests, training consisting of 42 trials was provided for every participant, where the participants received feedback via the tablet computer. In case of a wrong response, the correct source position was highlighted in the app. The objective of this training was to familiarize the subjects to the procedure as well as the stimuli used. Subjects were not allowed to search the presenting speaker by moving their heads during the stimulus presentations but could search it before giving their final answer. After training the participants performed at least 4 localization tests in an A-B-B-A paradigm. A total of 84 stimuli were presented in each localization test, meaning that each combination of speaker, level, and spectrum was presented twice. The subjects received no feedback during the tests. In the first test (A), the DL was programmed with a delay of 0 ms, thus representing the everyday device delay mismatch (+50 µs added by the microcontroller) of the participant. After this first localization test, the DL was set to $t_{\mathrm{Delay}}$ = *τ_HA_*, and another localization test (B) was conducted acutely. This test was conducted to determine whether the effects reported in Zirn et al. (2019) were acute or if a familiarization period to the changed device delay mismatch is required. In seven of nine participants, $t_{\mathrm{Delay}}$ was also set to *τ_HA_*–1 ms and *τ_HA_* + 1 ms in randomized order and tested acutely before familiarization.

After these acute tests, the DL was programmed with $t_{\mathrm{Delay}}$ that yielded the best localization results in the acute tests, that is, the combination of lowest rms error and lowest absolute signed bias, and the participants had a 1-hr familiarization period to adapt to the reduced delay mismatch. During this familiarization period of 1 hr, the participants went for a walk on the campus. They were instructed to pay attention to environmental sounds and to locate sound sources (e.g., birds) if possible. After this familiarization period, another localization test (B) was conducted to check for effects of familiarization and audiovisual training. Finally, the DL was programmed to $t_{\mathrm{Delay}}$ = 0 ms to test training effects over the course of the study. It should be noted that the participant Bim08 only conducted the first A and B test due to a hardware malfunction during the experiment (the battery charging unit was damaged and had to be replaced afterward). The entire measurement procedure, including the familiarization period and breaks when needed, took 3–4 hrs, consisting of a minimum of 378 trials for the subjects Bim201 and Bim202 and a maximum of 546 trials for all other subjects except Bim208.

### Evaluation and Statistical Analysis

The rms errors and signed bias of localization accuracy were calculated as proposed by Rakerd and Hartmann (1986). The rms error describes the discrepancy between the azimuth of a source and the azimuth of a subject’s response to that source corresponding to equation 1. Therefore, the rms error corresponds to the precision of the subject’s judgments according to ISO 5725 (International Organization for Standardization, 1994).

(1) $$\mathrm{rms}\;\mathrm{error}\;=\;A\sqrt{\frac{1}{M}\;\sum_{i=1}^{M}{\left(r_{i}-k_{i}\right)}^{2}}$$

(2) $$\mathrm{signed}\;\mathrm{bias}=\;\frac{A}{M}\sum_{i=1}^{M}\left(r_{i}-k_{i}\right)$$

A corresponds to the angle between two adjacent speakers (30° in the test setup used), *M* is the number of responses, *r_i_* is the response (1 to 7) on the *i*th trial, and *k_i_* is the number of the source on the *i*th trial. The reported rms error corresponds to the final calculation of the rms error after all 84 trials. The signed bias reflects the constant error or an error in trueness according to ISO5725 (International Organization for Standardization, 1994) in the listeners’ response. The signed bias can either be positive, indicating a bias of the listener to the right, or negative, indicating a bias toward the left.

Statistical analysis of the localization rms errors and signed bias of the test subjects included pairwise comparisons using Wilcoxon signed-rank tests with an alpha level of .05. Before statistical analysis, the signed bias for subjects having their CI on their left side was inverted. Therefore, a positive signed bias corresponds to a bias toward the CI.

## Results

### Best Delay for Device Delay Mismatch Reduction

**Figure 4.**
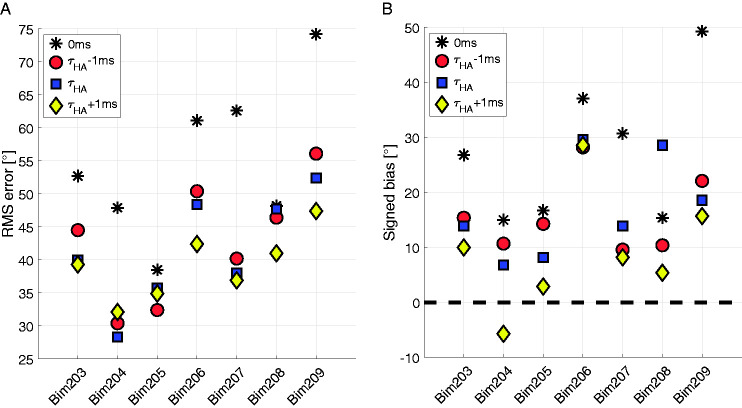
A: rms errors for three different delay values in acute sound source localization testing for subjects Bim203 to Bim209 in the first A and B conditions. B: signed bias for three different delay values in acute sound source localization testing for subjects Bim203 to Bim209 in the first A and B conditions. rms = root-mean-square.

Figure 4A and B shows the localization results for the seven participants that conducted localization tests with $t_{\mathrm{Delay}}$ set to *τ_HA,_ τ_HA_*–1ms and *τ_HA_*+1ms. Figure 4A shows the rms error for those seven participants, which instantaneously improved when the device delay mismatch was reduced. There was no clear tendency for which value for $t_{\mathrm{Delay}}$ yielded the best results.

In Figure 4B, the results for participants having their CI on the left side were inverted so that positive values always indicate a bias toward the CI. The dashed line represents zero bias which corresponds to perfect trueness in localization judgments. The data clearly showed a bias toward the CI that can be shifted toward 0° when the delay mismatch is compensated, for the values we tested; however, it reversed its sign only in one subject. When considering the signed bias, most patients had the best outcome with $t_{\mathrm{Delay}}$ set to *τ_HA_*+1ms. As the rms error was similar in the acute tests for each delay applied to CI stimulation compared with the initial condition without a CI delay, the value of $t_{\mathrm{Delay}}$ that yielded the lowest signed bias (i.e., the value closest to zero) was chosen and programmed into the DL for the following tests. An exception was Bim204 where *τ_HA_*+1ms led to a direction reversal, that is, a negative signed bias. Furthermore, in this case, the rms error was worse with *τ_HA_*+1ms compared with *τ_HA_*. Therefore, *τ_HA_* was programmed into the DL for further testing in case of Bim204.

### Sound Source Localization Accuracy

**Figure 5.**
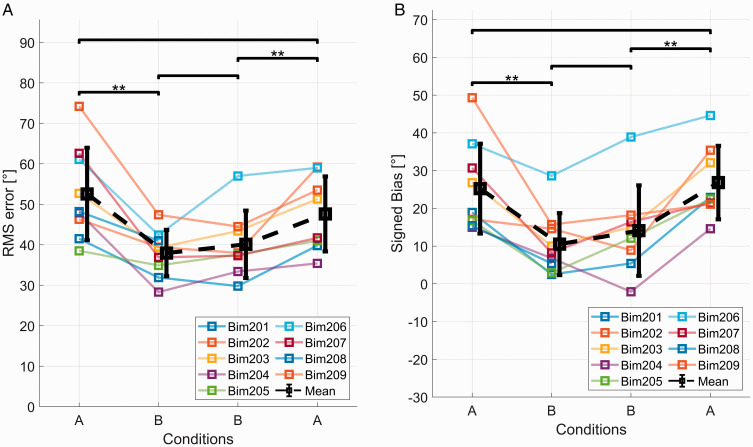
A: rms errors in the sound source localization test for nine participants in the A-B-B-A test design (significance levels: ** represents *p* ≤ .01). B: signed bias in the sound source localization test for nine participants in the A-B-B-A test design (significance levels: ** represents *p* ≤ .01). rms = root-mean-square.

Figure 5A and 5B shows the localization results for all nine participants in the A-B-B-A test design. The thick black line represents group means and standard deviations. In Figure 5b the data for participants wearing their CI on the left side have been inverted so that positive values always indicate a bias toward the CI. The average rms error and standard deviation in the initial condition was 52.6 ± 11.4°. The average rms error was 37.9 ± 5.7° when the device delay mismatch was reduced. After 1 hr of familiarization, the average rms error remained almost unchanged at 40.1 ± 8.3°. In the last condition (A), when the CI delay was removed, that is, the device delay mismatch set to the initial value, the mean rms error was at 47.6 ± 9.3°.

The mean signed bias was 25.2 ± 11.9° in the initial condition. After reduction of the device delay mismatch, the mean signed bias was 10.5 ± 8.2°. After 1 hr of familiarization to the reduced device delay mismatch, the mean signed bias was 14.1 ± 11.9°. When the initial device delay mismatch was restored, the average signed bias increased again to 26.8 ± 9.7°. Comparisons based on Wilcoxon signed-rank tests revealed that a reduction of the device delay mismatch led to a statistically significant instantaneous improvement of average –14.6 ± 8.5° in rms error (*p* < .01) and –14.7 ± 9.2° signed bias (*p* < .01). After 1 hr of familiarization, no further improvement could be shown in rms error with a mean difference and standard deviation of 2.6 ± 5.7° (*p* = .3) or signed bias with a mean difference and standard deviation of 2.9 ± 7° (*p* = .3). When the device delay mismatch was set to the initial condition, rms error and signed bias showed a statistically significant deterioration (*p* < .01). The mean difference for rms error was at 7.5 ± 6.4°, and the mean difference for the signed bias was at 12.7 ± 8.1°.

Because there was no significant difference between the two A conditions in rms error (*p* = .3, mean difference and standard deviation: 5.5 ± 11.7°) and signed bias (*p* = .7 mean difference and standard deviation: 0.4 ± 13.9), effects of procedural learning over the course of the experiments can be ruled out. All reported data are available on request.

**Figure 6.**
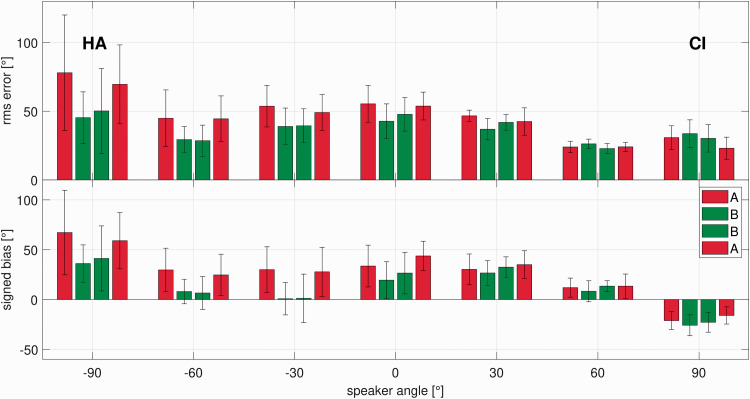
Speaker-Dependent Means and Standard Deviations for rms Error and Signed Bias for Nine Participants in the A-B-B-A Test Design. The data were inverted so the position of the CI is on the right ear in all participants. rms = root-mean-square; CI = cochlear implant; HA = hearing aid.

In Figure 6, angle-dependent results show that the rms error and signed bias before device delay mismatch reduction is highest on the HA side. The negative signed bias at 90° is most likely an edge effect, because the subjects could not input any speakers at more positive angles than 90°. The reduction of device delay mismatch led to an improvement of rms error and signed bias at almost every speaker position, being most prominent on the speaker at –90°, that is, the speaker directly on the HA side. For the speaker at 90°, a slight deterioration of rms error and signed bias could be observed.

## Discussion

In this study, the effect of the temporal adjustment of the CI processing delay and the HA processing delay was investigated in bimodal listeners. The outcomes show that (a) differences of the processing delays of hearing devices in bimodal listeners severely impair sound source localization in the horizontal plane and (b) this impairment can be mitigated at least partially by adding a simple DL to reduce the device delay mismatch. We found that the improvement in sound source localization is immediate, which is in line with a previous study (Zirn et al., 2019). No further improvement in localization accuracy was found after a familiarization period of 1 hr, but it is unclear if this 1-hr training was sufficient. Another outcome of the applied A-B-B-A test design is that effects of procedural learning can be ruled out, as the results deteriorated instantly in the second A condition to performance levels similar to the initial A condition.

Furthermore, this study shows that the reduction of the device delay mismatch improved not only the precision of the subjects’ judgments, expressed by the rms error, but also the trueness of the localization judgments expressed by the signed bias. This bias showed an orientation toward the faster modality (i.e., the CI) in all nine participants when the CI stimulation was not delayed. This indicates that a readjustment of sound localization is not achieved by neural plasticity even after several months or years of bimodal hearing (in our study, the mean bimodal experience was 3.8 years). Interestingly, sound localization accuracy improved instantly in all nine participants with all values of $t_{\mathrm{Delay}}$ , namely *τ_HA_* –*1 ms, τ_HA_* and *τ_HA_ +1 ms.* This further shows that even if the optimal setting of $t_{\mathrm{Delay}}$ is not yet determined, adjustment did not prove to be disadvantageous for any of the participants.

It should be noted that the device delay mismatch is frequency dependent (Zirn et al., 2015). Auditory brainstem response (ABR) Wave V delays match best at a frequency of 1 kHz, when the CI is delayed by the overall time delay of the HA *τ_HA_.* For 2 and 4 kHz, matching is better with $t_{\mathrm{Delay}}=\;$ *τ_HA_ +1 ms* and for 500 Hz with $t_{\mathrm{Delay}}=\;$ *τ_HA_* –*1 ms*.

The fact that all participating bimodal listeners benefitted from reducing the device delay mismatch is in line with the hypothesis that envelope ITD sensitivity improves with temporal alignment. Envelope ITD can be perceived across a wide frequency range by SSD CI users (Dirks et al., 2020) and at frequencies above 1 kHz by bimodal CI/HA users with sufficient temporal alignment of both modalities (Francart et al., 2009). Furthermore, improved temporal alignment in higher frequency regions by delaying the CI stimulation with *τ_HA_ +1 ms* may especially be helpful for ILD perception for those bimodal listeners who have considerable residual hearing at higher frequencies. This is in accordance with findings by Seebacher et al. (2019), who found that sound source localization precision in MED-EL CI users with SSD is highest when the CI stimulation is delayed by 1 ms on top of the processing delay reported by Zirn et al. (2015), resulting in an improved temporal matching of electric and acoustic hearing at higher frequencies in SSD CI users.

For bimodal listeners provided with other CI systems than those of MED-EL different CI delays have to be considered. Wess et al. (2017) reported a delay of the CI ear relative to a normal-hearing ear of 10.5–12.5 ms for Cochlear Ltd. and 9–11 ms for Advanced Bionics, which is considerably more than for CI systems of MED-EL. In such cases, the HA instead of the CI stimulation must be delayed to reduce the device delay mismatch. However, this approach has limitations, as HA processing latencies above 10 ms have been shown to cause subjective disturbances in patients (Agnew & Thornton, 2000; Bramsløw, 2010; Groth & Søndergaard, 2004).

Another, yet unknown, factor is how crucial a potential interaural tonotopic mismatch between modalities is for binaural processing. For example, in cases of incomplete insertion or for short electrode arrays, predominantly basal fibers are excited by the CI, whereas on the ear provided with the HA, often only the apical region of the cochlea is sufficiently stimulated. In envelope ITD detection tasks, it was found that a sufficient interaural match of excited characteristic frequencies is important (Bernstein et al., 2018; Dirks et al., 2020; Hu & Dietz, 2015). Furthermore, in bimodal listeners with pronounced residual hearing at higher frequencies (>1500 Hz), ILD may facilitate sound localization. In contrast to envelope ITD, ILD are presumably relatively robust against an interaural tonotopic mismatch as Francart and Wouters (2007) showed. In their experiments, ILD was usable for lateralization of sounds even for interaural frequency shifts up to 1 Octave. Kan et al. (2019) showed that shifts in interaural place of stimulation have high effects on the lateralization based on ITDs but lateralization based on ILDs was more robust in bilateral CI users. If the tonotopic alignment is found beneficial for binaural processing, this could be achieved by adjustment of the frequency allocation table in CI systems either by using postoperative imaging (Landsberger et al., 2015) or through psychoacoustic frequency matching techniques such as ITD discrimination (Bernstein et al., 2018; Hu & Dietz, 2015), interaural pitch comparison (Hu & Dietz, 2015), or sensitivity to binaural temporal envelope beats (Dirks et al., 2020). The optimization in the frequency domain just discussed, in combination with the optimization in the time domain (by the reduction of device mismatch), is a promising way to further improve bimodal hearing in the future.

## Conclusions

Our study shows that sound source localization improves in bimodal listeners when the temporal mismatch in the processing delays of HA and CI is reduced. A simple implementation with a frequency-independent DL on the CI side is already very effective. Because none of the nine test subjects showed a deterioration in localization accuracy in this study, we conclude that a temporal alignment between CI and HA by delaying the CI stimulation is a viable step to further improve bimodal provision.
